# Can the COPD web be used to promote self-management in patients with COPD in swedish primary care: a controlled pragmatic pilot trial with 3 month- and 12 month follow-up

**DOI:** 10.1080/02813432.2019.1569415

**Published:** 2019-01-31

**Authors:** Andre Nyberg, Malin Tistad, Karin Wadell

**Affiliations:** a Department of Community Medicine and Rehabilitation, Section of Physiotherapy, Umeå University, Umeå, Sweden;; b School of Education Health and Social studies, Dalarna University, Falun, Sweden

**Keywords:** COPD, physical activity, self-management, pragmatic trial, internet-based

## Abstract

**Objective:** Evaluate the feasibility of the COPD Web and its study design and study procedures and to increase the understanding of the potential effect of the tool in order to provide guidance for a future large scale trial.

**Design:** Parallel-group controlled pragmatic pilot trial.

**Subjects:** There was a total of 83 patients with COPD (mean age 70 ± 8 years with a forced expiratory volume in first second percent predicted of 60 ± 17%). The intervention group (*n* = 43) was introduced to and had access to the COPD Web in addition to usual care, while the control group (*n* = 40) received usual care alone.

**Main outcome measures:** The feasibility of the COPD Web (i.e., if and how the COPD Web was used) was automatically collected through the website, while outcomes on health, conceptual knowledge, and physical activity (PA) were collected through questionnaires at baseline, 3 months and 12 months.

**Results:** At 3 months, 77% of the intervention group was considered users, and the majority of time spent on the site was related to PA and exercises and was spent during the first month (>80%). In addition, the intervention group reported increased PA (odds ratio [OR] = 4.4, *P* < .001), increased conceptual knowledge in five domains (OR = 2.6–4.2, all *P* < .05), and altered disease management strategies (e.g., increased PA) (OR ≥ 2.7 *P* < .05) in comparison to the control group. The latter was also different between groups at 12 months (OR = 3.7, *P* = .044). Knowledge of PA was correlated with level of PA (*ρ* = .425–.512, *P* < .05) as well as to the use of PA as a strategy to manage their disease (*χ*^2^ = 11.2–32.9, *P* < .05).

**Conclusion:** Giving patients with COPD access to the COPD Web in addition to their ordinary primary care might be an effective shorter term (3 month) strategy to promote self-management. However, these results needs to be confirmed in a definitive large-scale trial.

**Key points**
Even though self-management strategies are an important part of chronic obstructive pulmonary disease (COPD) management, access to support for such strategies are limited for a large part of the COPD-population.

- Promoting self-management through the COPD Web might increase short-term levels of physical activity, promote conceptual knowledge and alter disease management strategies.
- The primary care COPD population in this study experienced limited impact of the disease in daily life, limited exertional dyspnea, and high generic quality-of-life, but vastly reduced levels of physical activity.
- A future large scale study should include strategies to encourage greater exposures to the COPD Web, including an extended analysis of factors associated with using or not using the tool over time and its impact on outcome measures, objective measures of conceptual knowledge, and physical activity, and it should include a large enough sample size to enable sub-group analyses and strategies to enhance recruitment.

## Introduction

Chronic obstructive pulmonary disease (COPD) is a major cause of morbidity and mortality, and with a steadily increase in prevalence, the disease is now the fourth leading cause of death worldwide [1]. The symptom burden of the disease, the impaired functional performance, and the decreased quality of life in patients with COPD are not only consequences of the underlying physiological disorder, but also dependent on the person’s ability to adapt to and to manage their disease [2,3]. Self-management strategies, including strategies to promote self-efficacy through increasing the patients’ knowledge and skills and their confidence in successfully managing their disease is therefore an important part of COPD management [2], and this is highly prioritized in Swedish treatment guidelines for this group of patients [4]. Support for self-management and education is often promoted by an asthma/COPD-nurse through pulmonary rehabilitation [4]. However, in Sweden only a limited proportion of patients with COPD get access to such services [5], which is related to both structural and individual barriers [6,7]. With regard to the former, limited access to pulmonary rehabilitation and to relevant health professionals have recently been reported in Swedish primary care settings [8,9]. For example, a survey from 2016 found that only 36% of patients treated within primary care in Sweden had met an asthma/COPD-nurse during the past year [9]. Furthermore, among patients with COPD, lack of knowledge and insight in their diagnosis, strenuous transportation and changing health have been identified as barriers for participation in pulmonary rehabilitation, thus reducing support for self-management strategies [6,10]. Consequently there is an urgent need to find new methods to facilitate the provision of self-management support to patients with COPD. Electronic health (eHealth) solutions are a promising way of delivering health services, and have previously been used as an alternative way of delivering pulmonary rehabilitation to patients with COPD [2]. However, even though eHealth solutions have been suggested to have the potential to deliver support for self-management in patients with COPD, effects are inconsistent and further research is warranted [11,12]. Therefore, to further address this question, our research group have developed the COPD Web, an internet based eHealth tool aimed at facilitating support for self-management for patients with COPD through increasing the patients’ knowledge and skills [13,14]. The COPD Web has been co-created together with health professionals (asthma/COPD-nurses, physicians, occupational therapists, dieticians and physiotherapists), patients with COPD and their relatives and experts in pulmonary rehabilitation [6,13]. However, before engaging on a definitive large-scale randomized controlled trial (RCT), conducting a pilot trial is highly recommended [15]. Thus, to provide guidance for a future definitive large-scale RCT, the objectives of this pilot trial were to evaluate the feasibility of the COPD Web, and its study design and study procedures as well as to increase the understanding of the potential effect of the tool with regard to aspects of health, knowledge, and PA [14].

## Methods and materials

### Study design

We conducted a parallel-group (1:1 allocation) controlled pragmatic pilot trial in line with the Consolidated Standards of Reporting Trials (CONSORT) statements for pragmatic trials and for pilot and feasibility trials [16,17]. The study is registered at ClinicalTrials.gov identifier: NCT02696187. Ethical approval was given by the Regional Ethical Board, Umeå University, Umeå, Sweden (Dnr: 2014-319-31, 2015-457-32). All patients gave written informed consent before enrolling in the study.

### Settings and participants

The pragmatic pilot trial took place at six primary care centers located in the middle and northern parts of Sweden. Three of the centers were situated in cities with 38,000–120,000 inhabitants, and the other three centers in sparsely populated areas with 2,000–4,500 inhabitants. The included centers were publicly funded, and all patients with a diagnosis of COPD (ICD-10:J44.9) who visited any of the included primary care centers from January 15–May 15, 2016, were eligible for inclusion in the intervention group while those patients who visited the centers from August 1 – December 30, 2015, were eligible for inclusion in the control group. Patients in the control group were identified from primary care units computerized records and were asked to participate by the study authors while the former group was asked to participate by health professionals. The different recruitment procedures were used to minimize contamination of the intervention to the control group (see the sample size, blinding, and randomization section below for further description). No specific exclusion criteria were utilized for patients or primary care centers.

### Intervention

**Figure 1.**
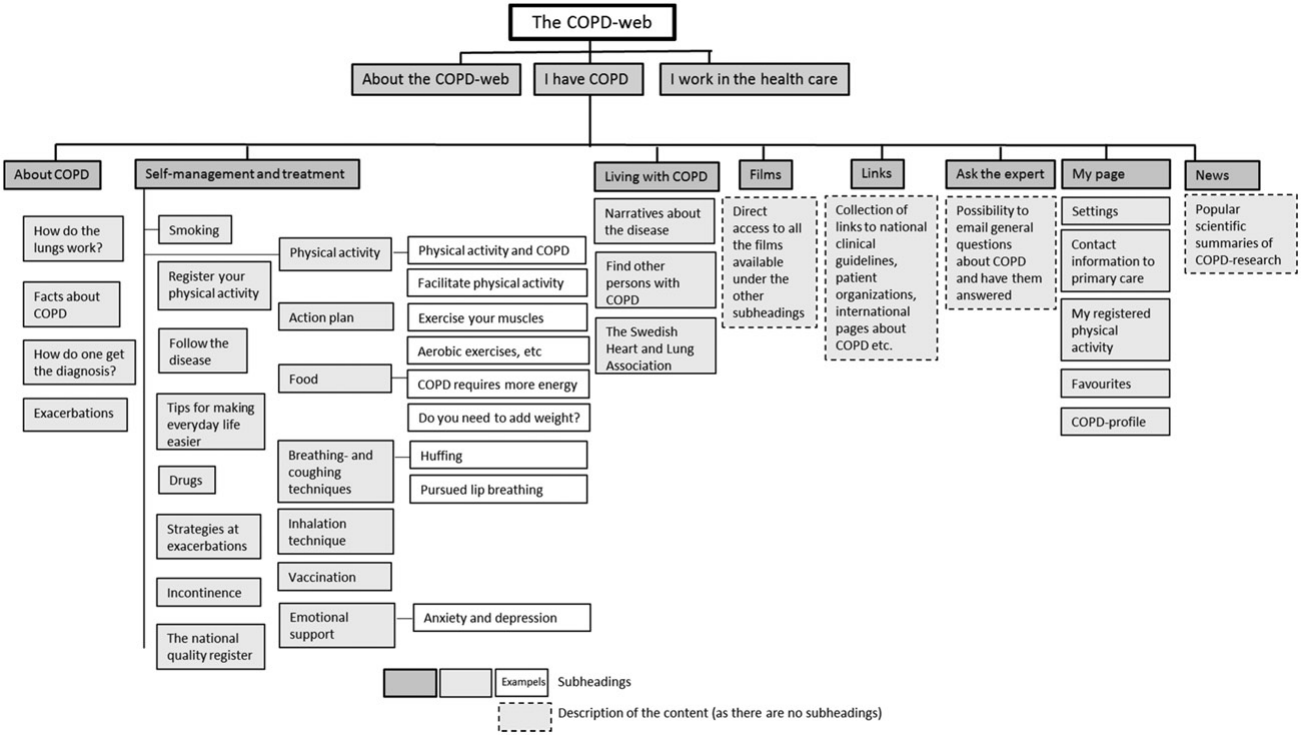
An overview of the content of the COPD-web with focus on the parts targeting patients with COPD [14]. Reprinted with permission from the indicated reference.

A detailed description of the COPD Web is available in the published protocol [14]. In brief, the COPD Web is an interactive web-page that was co-created with patients with COPD and their relatives, health professionals and experts in COPD management [6,13]. The COPD Web consists of two main sections – one directed at health professionals, and one directed at patients with COPD. An overview of the content of the COPD Web and, specifically the section on self-management for patients with COPD, is shown in Figure 1. The section for patients with COPD aims to support self-management by increasing the patients’ knowledge and skills [14]. The COPD Web includes, texts, pictures, and videos (e.g., how to perform exercise training, breathing techniques etc.) as well as interactive components such as a tool for registration of PA, including automated feedback. The content of the COPD Web is in line with the non-pharmacological health promotion interventions recommended by the Swedish National Board of Health and Welfare´s national guidelines for COPD management [4]. The COPD Web was introduced by the health professionals according to a pre-specified routine (Appendix, Box E1) [14]. No extra resources were provided to the primary care centers or the health professionals and the COPD Web was introduced to patients as a part of their ordinary work. Across the six primary care centers, seven health professionals (four asthma/COPD-nurses, one district nurse, one dietician and one physiotherapists with a mean work experience of 24 (SD 12) years) were involved in the study and were those who introduced the COPD Web to the intervention group. In addition, patients in the intervention group received a pedometer, instruction on how to use it, and an information sheet about the importance of PA.

### Comparator

Similar to what was given to the intervention group, patients in the control group received a pedometer, instruction on how to use it, and an information sheet about the importance of PA. Other than this, patients in the control group received usual care. In Sweden, the vast majority of patients with COPD are treated within primary care centers [4,9]. These include, but are not limited to, the use of long-acting anticholinergics and long-acting β2-agonists with 24 h duration, support for smoking cessation, support for PA and exercise, nutrition and education and support for self-management [4].

### Outcome measures

Patient-reported primary and secondary outcome measures were selected on the basis of disease specific clinical relevance, feasibility in a primary care center and relevance and feasibility in a Swedish context. A 3 month and 12 month follow-up period was selected because both are commonly used to investigate intervention-based effects in patients with COPD [18,19]. Outcomes on health, knowledge and PA were collected through questionnaires that were completed by the patients in their homes and were selected to increase our understanding of the potential effect of the tool. Feasibility of the COPD Web was operationalized as (1) time spent using the COPD Web and (2) which pages were used during the initial 3 month period, and this information was automatically collected through the website.

#### Primary outcome measure

Change in impact of COPD in daily life using the COPD Assessment Test (CAT) [20] was chosen as the primary outcome measure. This outcome was selected because it covers all of the symptomatic areas of COPD and because it has been shown to be responsive to healthcare interventions aimed to support self-management [21]. In addition, the CAT is a central part of the diagnosis of the disease [5], and is highly prioritized in Swedish guidelines for COPD management [4].

#### Secondary outcome measures

Investigation of the feasibility of the COPD Web was performed descriptively, analyzing if and how the COPD Web was used by the intervention group during the initial 3 months (frequency and time (minutes) spent on each different part of the COPD Web) (see Figure 1 for overview of content). Furthermore, change in health literacy was assessed using the Swedish Critical Health Literacy (C&CHL) scale [22]. Confidence in managing their COPD was assed using a standardized questionnaire specifically developed for this study. The questionnaire was pilot tested for face validity among experts in COPD management, health professionals and patients and adjusted accordingly prior to use in the study. The questions focused on the *importance* of different activities and the patient’s self-rated *knowledge* about these activities to manage their COPD, and the patients rated their response on a 5-point Likert scale (Appendix, Box E2). Patients also noted what they currently do to manage their COPD from a pre-specified list of ten activities/methods/measures (Appendix, Box E3). Aspects of PA were assessed using Grimby’s Activity Scale (a 6 point scale, with higher ratings meaning more active) [23], and indicators of PA and exercise as well as inactivity were retrieved from the Swedish National Board of Health and Welfare [24]. PA and exercise was rated on a scale ranging from 3, the lowest level of PA, to 18, the highest level of PA while inactivity (“How much do you sit during a normal day not counting sleep?") was rated on a 5 point Likert scale (higher score = less time spent sitting) [14]. Dyspnea was evaluated using the modified Medical Research Council (mMRC) [25] scale, self-efficacy to perform PA was assessed using the SCI Exercise Self-Efficacy Scale (ESES) [26], and generic quality of life (QoL) was assessed using the Swedish experience-based value set (tariff) for EQ-5D health states [27].

### Sample size, blinding and randomization

Because this was a pragmatic pilot trial, a sample size calculation was not performed. The final sample size in this trial was influenced by the total number of patients with COPD visiting any of the included primary care centers during the recruitment period. We estimated a maximal enrollment of 96 participants (around 16 per center) based on information provided by the primary care centers. Of importance, this pragmatic pilot study is part of a larger project that also aims to investigate the feasibility and effectiveness of the COPD Web among health professionals [14]. Because we anticipated that access to the COPD Web for health professionals (which was given prior to recruitment of patients started for this study) could affect how the health professionals interacted with the patients, the control group in the present study was recruited among patients who had visited the included primary care centers prior to the introduction of the COPD Web to the health professionals. However, even though patients in the intervention and control group were recruited from different samples (visits before *or* after the COPD Web was introduced to health professionals), data collection in the two groups was done in parallel using the same overlapping time frame (3 consecutive months). Nevertheless, the design of the pilot trial precluded randomization.

### Statistical analysis

Data analyses were intention-to-treat and were performed using generalized estimating equations. The linear response model was used for scale data, the ordinal logistic model was used for ordinal data and the binary logistic model was used for nominal data. Group and Time were set as factors, and primary care center was set as the covariate. Group*Time interaction was used for the analyses. Data at Baseline (M0), 3 months (M3), and 12 months (M12) are presented as the mean (standard deviation (SD)), median (interquartile range (IQR)), or percentage (%) depending on the distribution of the data. Between-group differences (M0 vs. M3, and M0 vs. M12) are presented as odds ratios [OR] and (95% confidence intervals (CI)) or Beta (B) and 95%CI, depending on the distribution of the data. A subgroup analysis (not initially planned [14]) was performed on outcomes related to PA in the intervention group in order to further explore the mechanisms associated with the observed effects. In addition, Spearman rank correlations (ρ) were used to analyze the correlation between PA-related outcomes and conceptual knowledge. The strengths of the correlation coefficients were categorized as low (0z0.25), moderate (0.25 > 0.50), strong (0.50 > 0.75), and very strong (>0.75). Pearson *χ*^2^ tests were used for correlations between the above mentioned PA variables and the activities the patients currently engage in to manage their disease (Box E3). No interim or additional analyses were made. No guideline for stopping the trial was utilized. The IBM Statistical Package for Social Sciences (SPSS) version 24 was used for data management and statistical analysis, and a *P* value of <.05 was considered statistically significant.

## Results

**Figure 2.**
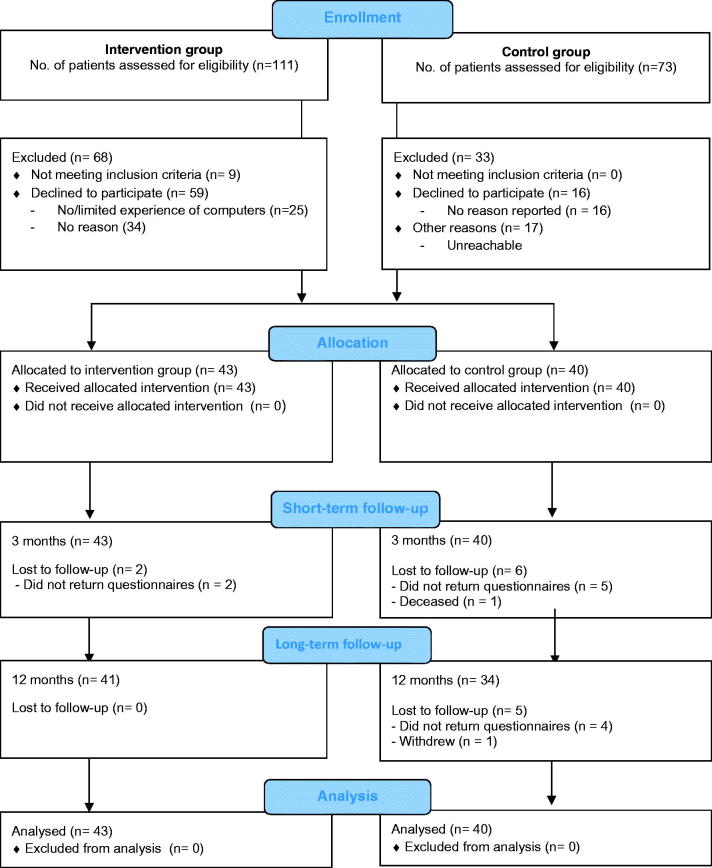
Flow of patients with COPD across the different stages of the trial.

All data collection in this pilot trial was performed between Jan 2016 and May 2017. Patient flow through the different stages of the trial is shown in Figure 2. Sociodemographic and baseline data of patients by group allocation are outlined in Table 1. Table 2 shows the results related to the impact of COPD in daily life, dyspnea, generic QoL, health literacy, and PA, while conceptual knowledge is shown in Table 3 and Table EI.

**Table 1.** Sociodemographic and baseline data of patients by group allocation.

	Intervention (*n* = 43)	Control (*n* = 40)
Age (years.)	65 (7)	71 (8)
Sex (Male)	23 (52%)	24 (60%)
FVC% predicted	81 (21)	76 (18)
FEV_1_ % predicted	60 (17)	59 (17)
FEV_1_/FVC	54 (10)	57 (10)
Stage of COPD^a^		
A (%)	24 (56%)	20 (51%)
B (%)	8 (19%)	9 (23%)
C (%)	5 (12%)	2 (5%)
D (%)	6 (14%)	9 (21%)
Smoking status		
Never smoker (%)	4 (9%)	2 (5%)
Ex-smoker (%)	29 (67%)	30 (75%)
Current smoker (%)	8 (19%)	8 (20%)
Pack years	28 (15)	32 (15)
Employment status		
Currently working (%)	10 (23%)	5 (13%)
Retired (%)	33 (67%)	34 (85%)
Sickness benefits (%)		1 (2%)
Living with		
Alone (%)	20 (47%)	23 (58%)
Family (%)	23 (53%)	17 (42%)
Education level		
Primary (%)	26 (60%)	22 (55%)
Secondary (%)	7 (17%)	10 (25%)
Tertiary (%)	10 (23%)	8 (20%)

Data is mean and standard deviation or percentages (%).

FVC: forced vital capacity; FEV_1_: Forced expiratory volume in 1 second.

a based on [28].

No significant differences were seen between groups (*p* > .05).

**Table 2.** Difference between groups on impact of COPD in daily life, dyspnea, generic quality of life, health literacy and PA.

	Data collection timepoints						GEE effects between groups - mean (95% CI), *P*-value	
	Month 0		Month 3		Month 12		Month 0 vs Month 3	Month 0 vs Month 12
Outcome	Exp (*n* = 43)	Con (*n* = 40)	Exp (*n* = 43)	Con (*n* = 40)	Exp (*n* = 43)	Con (*n* = 40)	Exp vs. Con	Exp vs. Con
Impact of COPD in daily life, dyspnea, generic quality of life and health literacy								
CAT	12.2 (5.7)	14.0 (6.0)	12.5 (6.1)	14.9 (6.3)	12.8 (7.2)	15.6 (7.7)	B = −0.7 (−2.6 to 1.2), *P* = 0.483	B = −1.1 (−2.2 to 4.3), *P* = 0.521
mMRC	1.45 (0.96)	1.64 (0.90)	1.23 (0.81)	1.61 (0.90)	1.36 (0.84)	1.62 (1.1)	B = −0.2 (−0.5 to 0.2), *P* = 0.283	B = −0.1 (−0.4 to 0.3), *P* = 0.709
EQ-5D	0.90 (0.09)	0.89 (0.07)	0.91 (0.09)	0.88 (0.09)	0.91 (0.08)	0.88 (0.11)	B = 0.02 (−0.02 to 0.06), *P* = 0.342	B = 0.02 (−0.03 to 0.07), *P* = 0.980
C&CHL	85.1%	76.9%	82.5%	76.5%	82.9%	66.7%	OR = 1.5 (0.5–4.9), *P* = 0.491	OR = 0.9 (0.3–3.2), *P* = 0.878
PA (PA)								
SoS-PA	9.1 (3.5)	9.2 (4.2)	10.8 (3.4)	8.6 (3.1)	9.7 (3.8)	8.7 (3.5)	B = 2.2 (0.6–3.9), *P* = 0.008	B = 1.1 (−0.8 to 3.0), *P* = 0.260
Grimby-PA	3 (2; 3)	3 (3; 3)	3 (3; 4)	3 (3; 3)	3 (3; 3)	3 (2.3; 3)	OR = 4.4 (1.9–10.4), *P* < 0.001	OR = 2.7 (0.9–7.8), *P* = 0.071
Inactivity^a^	5 (4.8;5)	4 (4;5)	5 (4;6)	5 (4;5)	5 (4; 5)	4 (4;5)	OR = 1.1 (0.6–2.1), *P* = 0.667	OR = 1.4 (0.7–2.7), *P* = 0.365
ESES	24.9 (1.1)	22.7 (1.3)	25.1 (1.1)	20.2 (1.2)	24.3 (1.3)	22.0 (1.2)	B = 3.1 (0.1–6.3), *P* = 0.059	B = 0.9 (−2.2 to 4.1), *P* = 0.554

Data is mean (SD), median (IQR 25;75) or percentage (%) of groups at 0, 3 and 12 months. General estimates equation (GEE) (beta/Odds ratio [OR] [95% confidence intervals], p-value) are presented for between group comparisons.

SD: standard deviation; IQR: interquartile range; Exp: intervention; Con: control; CAT: COPD Assessment Test; mMRC: modified Medical Research Council scale; EQ-5D: EuroQol five dimension scale; C&CHL: the Swedish Critical Health Literacy Scale; SoS: the Swedish National Board of Health and Welfare indicator questions for PA & exercise; ESES: Exercise Self-Efficacy Scale.

a Measured with the Swedish National Board of Health and Welfare scale indicator question for physical inactivity.

**Table 3.** Difference between groups on conceptual knowledge related to knowledge on how to take/perform/use different activities/measures to feel as good as possible in their disease.

	Data collection timepoints						GEE effects between groups - mean (95% CI), *P*-value	
	Month 0		Month 3		Month 12		Month 0 vs Month 3	Month 0 vs Month 12
Outcome	Exp (*n* = 43)	Con (*n* = 40)	Exp (*n* = 43)	Con (*n* = 40)	Exp (*n* = 43)	Con (*n* = 40)	Exp vs. Con	Exp vs Con
Conceptual knowledge: I have sufficient knowledge to affect/take/perform/use								
My health and wellbeing in my COPD	4 (3; 5)	4 (3; 5)	4 (3; 5)	4 (3; 4)	4 (3; 5)	4 (3; 4)	OR = 2.8 (1.1 to 6.9) *P* = .028	OR = 2.9 (0.9 to 8.6) *P* = .062
Daily physical activities	4 (3; 5)	4 (3; 5)	4 (4; 5)	4 (3; 5)	4 (3; 5)	4 (3; 5)	OR = 2.9 (1.3–6.3) *P* = .007	OR = 2.0 (0.8–5.1) *P* = .158
Exercise training	4 (3; 4)	4 (3; 5)	4 (3.3; 5)	3 (2.8; 4)	4 (3; 4.8)	3.41 (3; 4)	OR = 4.2 (1.7 to 10.5) *P* = .002	OR = 1.8 (0.7–4.8) *P* = .260
Breathing techniques at rest	3 (2; 4)	3 (1; 4)	4 (2; 4)	3 (1.8; 4)	3 (2; 5)	3 (2; 4)	OR = 2.6 (1.1–6.6) *P* = .038	OR = 1.8 (0.6–5.1) *P* = .302
Breathing techniques during activity/exercise	3 (2; 4)	3 (1.8; 4)	4 (2; 4)	3 (2; 4)	3 (2; 5)	3 (2; 4)	OR = 2.5 (0.9 to 6.7) *P* = .066	OR = 2.1 (0.7 to 6.5) *P* = .186
Techniques for mucus mobilization	2 (1; 3)	3 (1; 4)	3 (2; 4)	2.5 (1.8; 3)	3 (1; 4)	3 (2; 4)	OR = 3.2 (1.1 to 9.1) *P* = .029	OR = 1.6 (0.5–5.1) *P* = .419
Energy conservation techniques	3 (2; 4)	3 (1; 4)	3 (3; 4.3)	3 (2; 4)	4 (3; 4)	3 (1; 4)	OR = 1.2 (0.4–3.4) *P* = .725	OR = 1.4 (0.5–4.1) *P* = 0.549
Food adapted for my condition	2.5 (2; 4)	3 (1; 4)	3 (2; 5)	3 (2; 4)	4 (2; 5)	3 (2; 4)	OR = 1.6 (0.7–3.9) *P* = .282	OR = 2.3 (0.96–5.7) *P* = .061
Medication in accordance to ordination	5 (4; 5)	5 (4; 5)	5 (4; 5)	5 (4; 5)	5 (4; 5)	5 (4; 5)	OR = 1.5 (0.6–3.5) *P* = .365	OR = 1.3 (0.5–3.3) *P* = .626
Medication with accurate inhaler techniques	5 (3.8; 5)	5 (4; 5)	5 (4; 5)	5 (3.8; 5)	5 (3; 5)	5 (4; 5)	OR = 0.9 (0.4 = 2.2) *P* = .853	OR = 0.7 (0.3–1.9) *P* = .464
Contact health care if having symptoms for exacerbations	4.5 (3; 5)	5 (4; 5)	4 (3; 5)	4 (3; 4)	4 (3; 5)	5 (4; 5)	OR = 2.6 (0.8–8.0) *P* = .095	OR = 0.9 (0.3–2.8) *P* = .868

Data is mean (SD), median (IQR 25;75) or percentage (%) of groups at 0, 3 and 12 months. General estimates equation (GEE) (beta/Odds ratio [OR] [95% confidence intervals], p-value) are presented for between group comparisons.

SD: standard deviation; IQR: interquartile range; Exp: intervention; Con: control.

With regard to if and how the COPD Web was used (feasibility of the tool), 95% of all patients with COPD enrolled into the intervention group created an account and visited the site at least once. However, only 77% (*n* = 33) of the participants were considered users and thus spent time on the *different* pages on the COPD Web. The mean number of visits among users was 5.1 (SD 6.2) visits, spending a mean of 10.6 (SD 9.5) minutes per visit. Participants visited on average 11 (SD 10) different sub-pages per visit to the COPD Web. Of the total amount of time spent on the site across these sub-pages (see Figure 1 for overview of content), 54% was spent on pages related to self-management and treatment with pages targeting PA and exercise accounting for the vast majority of this time (44% of the total time spent on the COPD Web). In addition, 28% of the total time was spent on disease specific pages containing information about COPD (e.g. facts about COPD, exacerbations, how the lungs work, etc.), 12% was spent on the video section, and 6% was spent on site-specific functions such as links, ask the expert, my page, and news. The vast majority of time spent on the COPD Web was spent during the first month, and this decreased over time (Month 1: 82% of total time spent on the site, Month 2: 10% of total time spent on the site, Month 3: 8% of total time spent on the site).

Despite the modest use of the COPD Web, 3 month effects in favor of the intervention group were found with regard to PA as measured with the Grimby’s Activity Scale [23] and with the Swedish National Board of Health and Welfare indicator questions about PA [24] (Table 2), with regard to conceptual knowledge in five domains (Table 3), and with regard to increased self-reported use of accurate inhaler techniques while taking medication and increased self-reported use of PA as strategies to manage their disease. The latter was also significantly different between groups at 12 months (Table 4). Other than a significant effect on conceptual knowledge and *Q8)* “knowledge on how to eat food adapted for my condition” and *Q10* “knowledge on how to take medication with accurate techniques” (*P* < .05 at both 3 and 12 months), effects were similar across primary care centers.

**Table 4.** Difference between groups on activities/methods/measures patients currently do to manage their disease.

	Data collection timepoints						GEE effects between groups - mean (95% CI), *P* -value	
	Month 0		Month 3		Month 12		Month 0 vs Month 3	Month 0 vs Month 12
Outcome	Exp (*n* = 43)	Con (*n* = 40)	Exp (*n* = 43)	Con (*n* = 40)	Exp (*n* = 43)	Con (*n* = 40)		
To manage my COPD I currently								
Am physical active in my daily life	84.2%	89.7%	92.3%	76.5%	85.4%	72.4%	OR = 6.3 (1.2–33.1) *P* = .030	OR = 3.7 (1.0–13.4) *P* = 0.044
Perform physical exercise at least once a week	39.5%	43.6%	46.2%	35.3%	48,8%	44,8%	OR = 1.9 (0.8–4.5) *P* = .158	OR = 1.4 (0.5–3.9) *P* = .493
Use breathing technique’s at rest	23.7%	35.9%	33,3%	29.4%	41.5%	34.5%	OR = 2.2 (0.9–5.4) *P* = .097	OR = 2.4 (0.8–7.4) *P* = .116
Use breathing technique’s during ADL	23.7%	25.6%	30.8%	29.4%	39.0%	31.0%	OR = 1.2 (0.4–3.3) *P* = 0.744	OR = 1.6 (0.5–4.9) *P* = 0.417
Use techniques for mucus elimination	15.8%	17.9%	23.1%	23.5%	19.5%	24.1%	OR = 1.1 (0.3–4.1) *P* = .844	OR = 0.9 (0.3–3.1) *P* = .852
Use energy conservation techniques in my daily life	39.5%	33.3%	43.6%	52.9%	51.2%	37.9%	OR = 0.5 (0.2–1.4) *P* = .192	OR = 1.3 (0.5–3.3) *P* = 0.557
Eat food adapted to my condition.	15.8%	7.7%	15.4%	8.8%	19.5%	10.3%	OR = 0.8 (0.2–4.6) *P* = .835	OR = 0.9 (0.2–5.1) *P* = 0.939
Take medication in accordance to ordination.	92.1%	89.7%	97.4%	88.2%	95.1%	89.7%	OR = 3.8 (0.5–27.1) *P* = 0.179	OR = 1.7 (0.3–8.6) *P* = 0.518
Take medication with accurate techniques.	68.4%	71.8%	82.1%	67.6%	65.9%	82.8%	OR = 2.7 (1.1–6.6) *P* = .026	OR = 0.5 (0.1–1.7) *P* = 0.255
Contact health services if experiencing symptoms for exacerbations	57.9%	64.1%	76.9%	67.6%	61.0%	69.0%	OR = 2.1 (0.7–6.2) *P* = .181	OR = 0.9 (0.3–2.5) *P* = .876

Data is mean (SD), median (IQR 25;75) or percentage (%) of groups at 0, 3 and 12 months. General estimates equation (GEE) (beta/Odds ratio [OR] [95% confidence intervals], p-value) are presented for between group comparisons.

SD: standard deviation; IQR: interquartile range; Exp: intervention; Con: control.

The subgroup analysis on PA within the intervention group between those who used (*n* = 33) and those who did not use (*n* = 10) the COPD Web revealed significantly larger effects on PA in the former group (B = 3.286 (0.282–6.291, *P* = .032). In addition, significant correlations were found across all time points between self-reported PA and knowledge on how to *Q1)* “affect my health and wellbeing in my COPD” (*ρ* = .228–.378, *P* < .05), *Q2)* “perform daily physical activities” (*ρ* = .425–.512, *P* < .05) and *Q3)* “perform exercise training” (*ρ* = .282–.400, *P* < .05). Significant correlations were also seen during both 3 month and 12 month follow-up between using PA as a strategy to manage their disease and knowledge on how to be physically active (M0: *χ*^2^ = 4.1, *P* = .391, M3: *χ*^2^ = 11.0, *P* = .012, M12: *χ*^2^ = 11.4, *P* = .023) as well as regarding level of PA (*χ*^2^ = 11.2–32.9, *P* < .05 across all time points).

## Discussion

The objectives of this pragmatic pilot trial were to provide guidance for a future definitive large scale RCT, by (1) evaluating the feasibility of the COPD Web, (2) evaluating the feasibility of the study design and study procedures and (3) increasing the understanding of the potential effect of the tool with regard to aspects of health, knowledge and PA [14]. The primary findings are that (1) approximately 3 out of 4 patients with COPD in the intervention group used the COPD Web during the initial 3 months, a with a large part of the time spent on pages related to PA and exercise with the vast majority (>80%) of the time spent on the site being during the initial month. We also found (2) that neither impact of COPD in daily life, dyspnea, generic QoL or health literacy was highly reduced in patients with mainly moderate COPD currently registered at primary care centers in Sweden, thus indicating that other more relevant outcomes (e.g., PA which was vastly reduced) should be targeted in a future trial. Finally, (3) with regard to the potential effect of the COPD Web, pilot findings indicate that providing access to the COPD Web to patients with COPD currently registered within Swedish primary care settings seem be an effective short-term (3 month) strategy to increase the level of PA, increase conceptual knowledge, and to alter the strategies used by the patients to manage their disease compared to usual care. How the findings from this pragmatic pilot trial will inform a future large-scale study are discussed in the sections below and summarized in the *implications for future large-scale RCT* section (end of discussion).

### Strengths and weaknesses

The pragmatic design is a strength of this pilot trial. Except for an introduction of the COPD Web, no additional support was provided to either the primary care centers or the health professionals, and the intervention was delivered as a part of their ordinary work. Another strength was that this trial was conducted at six primary care centers in Sweden, and despite a relatively small sample of centers, the included centers had different geographical locations, were located in differently populated areas and were all publically funded, the latter being a characteristic of the vast majority of primary care centers in Sweden. In addition, the included patients with COPD were not selected based on rigorous inclusion and exclusion criteria, and all of these factors increase the external validity and generalizability of the trial findings. However, the lack of other inclusion and exclusion criteria became a limitation of the present trial because the baseline scores on several of our selected outcome measures gave limited room for improvement in either the intervention or the control group. Even though several of the included outcome measures did not increase over the 12 month follow-up period, it is important to note that they did not decrease in either of the groups which, which is especially relevant considering that COPD is a disease that worsens over time [5]. Another limitation related to the design of this pragmatic pilot trial was that patients were recruited during a pre-specified fixed time frame, which resulted in the final sample size not being known prior to deciding on inclusion of outcome measures. However, with regard to our primary outcome measure, the CAT, the final sample size of this pilot trial was slightly larger (83 vs. 73) than a previous study in which a nurse-led educational telephone intervention was used to support self-management in COPD and in which a significant effect on the CAT was found [21]. This suggests that the pilot trial sample size should have been large enough to be able to detect changes on the CAT. Furthermore, even though sample size is of importance and results, especially non-significant results, could be misleading if the sample size is too small (an underpowered trial), it is not certain that an increase in sample size would change these results [29]. Moreover, due to technical difficulties, use of the COPD Web could only be monitored and registered during the initial 3 months, making definitive conclusions on how use (or lack of use) of the COPD contributed to the lack of effects at 12 months. Lastly, different recruitment strategies were used for the intervention and the control groups, a decision that was taken to minimize the risk of contamination between groups because all health professionals had access to the COPD Web as part of their clinical work prior to the recruitment of patients to this pilot trial. However, this precluded randomization and is a limitation of the present trial and the non-randomized approach used in this pilot trial is the largest difference between the pilot trial and a planned future large-scale study.

### Interpretation of findings in relation to previously published work

#### Effects on impact of COPD in daily life, dyspnea, generic QoL and health literacy

Concerning the impact of COPD in daily life, dyspnea, generic QoL, and health literacy, we did not observe any within or between-group differences. In contrast to our findings, a nurse-led educational telephone intervention aimed to support self-management in patients with COPD in primary care was previously found to be more effective than usual care with regard to reducing the impact of COPD in daily life [21]. That trial included pre-determined contacts between the patients and an advanced nurse practitioner throughout the 6 week intervention period [21]. The use of additional support for self-management programs, such as monthly telephone support or home-care visits, has also been found to be effective with regard to disease-specific QoL [30]. This suggest that the use of eHealth tools, such as the COPD Web, might not be enough if the goal is to increase self-management related to similar outcomes [31]. Additional support over time; for example, from health professionals, might be necessary to increase the effects [21,30], even though this is not a universal finding [32]. However, the lack of effect in the present trial could also be a result of a selection bias; for example, baseline CAT scores in the intervention group were 12.2 out of 40, with <10 indicating a low impact of COPD in daily life. The mean score of 12.2 in our sample of mainly GOLD II patients with COPD was even lower than the mean score of 13.7 reported by patients with mild COPD (GOLD I) in a systematic review of the CAT published in 2014 [33]. Patients also had a low mean baseline mMRC score (1.45), high baseline health literacy (86% had no limitation) [22], and high baseline generic-QoL (tariff 0.90), the latter being higher than the mean tariff (0.88) seen in a sample of the Swedish general population [34], and thus there was little room for improvement in these outcomes, which likely contributed to the lack of effects due to a ceiling effect. Similar to these findings, Bischoff et al. [35] did not show any long term benefits in terms of quality of life over usual care alone in patients with COPD in general practice when comparing comprehensive self-management to routine monitoring. The combination of a population scew towards milder COPD cases in combination insufficient evidence of interventions in more mild COPD [36] could make it difficult to demonstrate effects in intervention studies in primary health care. Nevertheless, because one of the objectives of this pragmatic pilot trial was to investigate the feasibility of the study design and procedures in order to provide guidance for a future large scale RCT these findings are of importance. Because the impact of COPD in daily life, dyspnea, generic QoL, and health literacy do not seem to be highly reduced in patients with mainly moderate COPD currently registered at primary care centers in Sweden, these findings suggest that a future large-scale RCT should focus its attention on other, more relevant outcomes for this part of the COPD-population and/or increase the sample size to enable sub-group analyses based on baseline values.

#### Effect on PA and inactivity

Despite limited impact of COPD in daily life, low dyspnea scores and high generic QoL, the level of PA was highly reduced in our sample. For example, only 24% of our total study sample (30% of the intervention group) met the national recommendations of at least 150 minutes of moderate activity per week [24]. Thus implicating that PA level might be a clinically relevant target for self-management strategies in patients with COPD within the primary care system in Sweden and which preferably should be prioritized in a future trial. In comparison to the usual care group, the intervention group reported an increase in the amount of daily PA as well as an increased amount of time spent performing exercise training at 3 months (at 3 months, 53% of the intervention group reached the national requirements compared to 18% in the control group, *P* < .001 between groups) (Table 2). However, at 12 months, even though 42% of the intervention group still met the national requirements (18% in the control group), this difference was not significantly different between groups compared to baseline data (Table 2). Similar results were seen on Grimby’s Activity Scale [23] in which the odds of increasing PA at 3 months in the intervention group were over 3 times as high compared to the control group. A similar trend was also seen at 12 months (≥ 2 higher odds), even though this was not statistically significant (*P* = .079). In contrast to our findings, neither Voncken-Brewster et al. [12] nor Vorrink et al. [37] found an effect on PA when comparing usual care with eHealth self-management interventions (a website alone or in combination with a mobile phone app). However, similar to our findings, Jolly et al. [19] found shorter-term effects (6 months) but not 12 month effects on the level of PA using telephone health coaching to support self-management in people with COPD within primary care in the UK. Overall, these pilot data provide support for further exploration of the potential effect of the COPD Web on PA, which preferably should include an objective measure of PA because the present study only included subjective measures that could be biased by an overestimation of individual PA as well as the rely on recall of the patients [38]. Objective measures were intended in the present pilot trial [14], but only a very limited number of patients had such measures taken (<16%), which precluded statistical comparisons.

#### Effects on COPD-specific conceptual knowledge

An important part of self-management in patients with COPD is to increase the patients’ knowledge and skills and their confidence in successfully managing their disease [2]. Self-management strategies have previously been shown to increase COPD knowledge in patients with COPD as measured by the Bristol COPD knowledge questionnaire (BCKQ) [39,40]. The BCKQ includes questions on COPD specific knowledge with regard to topics such as symptoms, breathlessness, exercise, smoking, etc. [41]. However, because the questionnaire is unavailable in Swedish, a standardized questionnaire specifically developed for this study was utilized. The different topics were selected based on the Swedish National Board of Health and Welfare guidelines [4] and included topics similar to the BCKQ questionnaire [41]. In comparison to the control group, the intervention group demonstrated self-reported increases in conceptual knowledge in several domains, e.g., increased knowledge on how to perform daily physical activities and how to perform exercise training at 3 months which, therefore indicated that the COPD Web, in a similar way as other self-management strategies, could be used to increase conceptual knowledge [39,40]. However, it is important to note that the questionnaire focused on whether the patients themselves thought that they had increased their knowledge. An objective measure of conceptual knowledge, similar to the BCKQ [41], should be considered in a large-scale trial. Furthermore, at 3 months the intervention group reported to a larger extent than the control group that they “are physically active” and “take medications with accurate techniques” as strategies to manage their disease. However, especially with regard to the latter, these findings should be interpreted with caution because discrepancy between the patient’s and an expert’s perception of accurate inhaler technique is common, and patients often overestimate their ability [42].

#### Feasibility of the COPD web

The analysis of the feasibility of the COPD Web in this pilot trial primarily focused on if and how the COPD Web was used during the initial 3 month period. During this time, 77% of patients having access to the COPD Web were considered users, and the majority of time on the site was spent on pages related to PA and exercise and was spent during the first month (>80%). Total time on the site was, on average, 45 min, which was surprisingly low and indicate that a future trial should include strategies to enhance use of the tool. One such alternative could be to incorporate a push-notifications function (e.g., containing reminders to access the page) in the COPD Web because this approach has recently been found to encourage greater exposures to e-health interventions without deterring engagement [43]. However, even though the average time spent on the COPD Web was low, the amount of time might not be the best predictive factor for our outcomes. For example, we know from studies on adherence to medication that this varies vastly across patients and that some adhere directly when receiving information/instructions while others might require multiple interventions before adhering [44]. In addition, <2 h of brief education has previously been shown to increase conceptual knowledge (according to the BCKQ) in people with COPD within primary care in Canada [45]. However, in addition to gaining access to the COPD Web, patients in the intervention group were also in contact with health professionals who have had access to the COPD Web as part of their clinical work [14]. Thus, it could be difficult to determine if the observed effect on PA in the intervention group is primarily due to the patients’ access and use of the COPD Web (which was modest), a potentially changed behavior of the health professionals (e.g., possibly giving more advice/information on the importance of PA), or a combination of the two. Considering the patients’ modest use of the COPD Web throughout the initial 3 months, a subgroup analysis, which was not initially planned [14], was performed on patients in the intervention group, and this revealed that larger effects on PA were seen among those patients who used the COPD Web compared to those who did not use the COPD Web. This suggest, that use of the COPD Web itself, and not just the combination of information provided by the health professionals together with the COPD Web, seems to be beneficial if the goal is to improve level of PA, at least in the shorter term (3 months). However, even though the information provided by health professionals in the present pilot trial did not seem to affect the study results, the role of health professionals should not be neglected in a future trial because the quality of the information provided by the health professionals might vary. For example, previous research has found that patients at primary healthcare centers that are led by a disease-specialist primary care nurse (i.e. an asthma/COPD-nurse) experience fewer COPD exacerbations as well as fewer hospitalizations [46] than patients enrolled at primary care centers that do not have an asthma/COPD nurse. Results that also have been supported by others nationally as well as internationally [47–49] suggest that the type of primary care center (and access to different health professionals) should be considered in a future trial.

Over the course of the 12 month intervention period, no harms or unintended effects on individual patients with COPD were reported. Recruitment rates and baseline characteristics were also similar between groups. However, even though only having a COPD diagnosis was sufficient to be included in the present trial, around 50% of potentially eligible patients declined participation. Not having a computer or having limited experience with computers were the main reasons for declining to participate, highlighting that an Internet-based tool to facilitate self-management is not for everyone. However, this was never the intent of the tool. In contrast, the numbers could be seen from the other perspective, indicating that an Internet-based tool could be a feasible and seemingly effective way of providing self-management to increase PA as well as conceptual knowledge in almost half of the patients with COPD within primary care. In addition, roughly 50% of the participants in the usual care group also declined to participate, even though their participation had no active intervention other than filling out and submitting questionnaires at two time points. No sub-group analysis of those who accepted and those who declined in the two groups were possible, so we cannot elucidate on potential factors. Our rate of inclusion was similar to another pragmatic self-management trial performed in patients with COPD in which 40% of 291 potentially relevant patients accepted inclusion [50]. This highlight the difficulty of including individuals in this type of pragmatic trials. Nevertheless, these findings indicate that strategies to increase recruitment rates should be considered in a larger trial.

#### Mechanism of effects

From our additional analyses, we learned that knowledge on how to affect their health and wellbeing in relation to their disease, how to perform daily PA, and how perform exercise training were positively moderately to strongly correlated to the amount of self-reported PA across all time points as well as moderately correlated to using PA as a strategy to manage their disease at both 3 month and 12 month follow-up. This suggest a link between knowledge of PA and the amount of PA performed in which greater knowledge on *how* to be active was associated with *being* more active. With this in consideration, the proposed mechanisms behind using the COPD Web as a tool to deliver support for self-management strategies to people with COPD is that increasing access to (and use of) COPD-specific information on a specific topic could result in increased knowledge on that topic, which in turn, could lead to changes in outcomes related the topic in which the knowledge has increased. Based on our pilot data, the large part of time spent on the COPD Web on pages related to PA (44%), the increased knowledge related to how to be physically active, and the increased use of being physically active in their daily life as a strategy to manage their disease might explain *how* use of the COPD Web resulted in the observed increases in PA found in favor of the intervention group at the 3 month follow-up. However, such a potential link needs to be confirmed, and sufficient sample sizes for subgroup analyses to further explore this link should be a goal of a future trial. Nevertheless, similar findings as seen in this pilot trial have previously been highlighted in other populations; for example, knowledge of PA recommendations has been associated with higher stages and levels of PA behavior, and a brief educational exposure to PA recommendations led to improved levels of PA behavior in young adults [51]. However, whether similar results would be seen among patients with more severe disease or in other contexts (e.g., within home health care) remains to be determined. It could be that the relevance of disease specific self-management strategies are lower in patients with more complex health and social needs in which a more holistic approach might be a more applicable approach [52].

### Implications for a future large-scale RCT

What we learned from this pragmatic pilot trial that will inform the design of a large-scale study is as follows.

- The COPD Web seems to be an effective shorter term (3 month) strategy to increase self-reported PA, and conceptual knowledge and to alter disease management strategies.
- In patients with mainly moderate COPD currently registered at primary care centers in Sweden neither impact of COPD in daily life, dyspnea, generic QoL or health literacy seem to be highly reduced, thus highlighting that other outcomes should be prioritized. Irrespective of this, the level of PA was vastly reduced in this group and should be a key outcome in a future large-scale study, which should include objective measurements of PA.
- Analysis of the use of the COPD Web throughout the full intervention period is required in order to inform on the link between use of the Internet-based tool and any possible effect.
- An extended analysis on factors associated with using or not using the COPD Web over time is needed, and strategies to enhance adherence and means/methods to promote and support relevant self-management strategies among patients with COPD are warranted.
- Assessment of COPD-related knowledge should include an objective measurement of conceptual knowledge rather than be self-reported.
- The sample size should be large enough to enable sub-group analyses and to account for the design effect.
- Recruitment of patients should be done in a way to allow for randomization.
- Strategies to enhance recruitment rates should be incorporated.

Of importance, the lessons learnt from this pilot trial are not only relevant for a future large-scale trial targeting the COPD Web, several of these could be generalized to other studies, especially studies investigating the effect of eHealth solutions. For example, the importance of analyzing factors between users and non-users of the eHealth solution in order to find out for whom these type of interventions are suitable.
